# SARS-CoV-2 Infection (COVID-19) and Herpes Simplex Virus-1 Conjunctivitis: Concurrent Viral Infections or a Cause-Effect Result?

**DOI:** 10.7759/cureus.12592

**Published:** 2021-01-09

**Authors:** Jarelys M Hernandez, Hareesh Singam, Abida Babu, Sadaf Aslam, Seetha Lakshmi

**Affiliations:** 1 Infectious Diseases, University of South Florida Morsani College of Medicine, Tampa, USA; 2 Internal Medicine, University of South Florida, Tampa, USA

**Keywords:** covid-19, sars-cov-2, conjunctivitis, hsv-1

## Abstract

The pulmonary effects of severe acute respiratory syndrome coronavirus-2 (SARS-CoV-2), the virus that causes coronavirus disease (COVID-19), are well documented; however, more evidence is needed to understand its effect on multiple organ systems. We present the case of a 69-year-old male with dyspnea for two weeks and bilateral conjunctivitis who tested positive for SARS-CoV-2. He was found to be hypoxic, requiring supplemental oxygen. On hospital day two, he complained of worsening left eye pain with the development of a left lower eyelid ulcer. He underwent a CT of facial bones, which showed findings consistent with pre-septal cellulitis and abscess. Samples from bilateral conjunctival secretions and left lower eyelid ulcer tested positive for herpes simplex virus-1 (HSV-1), and negative for SARS-CoV-2. He received supportive care, antibiotics, and famciclovir with almost complete resolution of his ocular complaints. This case illustrates an atypical COVID-19 presentation and raises concern as to how this virus modulates the immune system, allowing for concurrent viral infections.

## Introduction

Coronavirus disease 2019 (COVID-19) is primarily a respiratory infection; however, it is now known to affect multiple organs, including the eye [1]. Due to the novelty of the virus, there are scarce data on COVID-19-related ocular infections in the medical literature. One study conducted in China noted that 31.6% of COVID-19-infected patients had ocular manifestations consistent with conjunctivitis [2]. However, the initial presentation of COVID-19 being an ocular manifestation seems rare. Other viruses such as herpes simplex are known to commonly present with ocular manifestations, with the latter having an annual incidence of 11.8 new cases for every 100,000 population [3]. In fact, the reactivation of latent herpes simplex virus (HSV) in the sensory ganglia may lead to initial or recurrent disease, typically monocular [3].

## Case presentation

A 69-year-old Caucasian male with a medical history of type II diabetes mellitus, coronary artery disease, peripheral arterial disease, and stage III squamous cell lung cancer receiving weekly docetaxel presented to the ER in March 2020 with complaints of progressive dyspnea, cough, and scant white sputum for 14 days. He was a personal care aide who worked in a rehabilitation facility where multiple staff and patients had tested positive for COVID-19. Upon presentation, he was afebrile, normotensive, and hypoxemic, requiring 2 liters of oxygen via nasal cannula. He also reported bilateral eye pain, more significant in the left eye than the right, with associated bilateral conjunctival erythema, pruritus, chemosis, and foreign body sensation. He denied any blurry vision, changes in visual acuity, or previous ocular problems. Of note, the patient was enrolled in a double-blinded, randomized placebo-controlled trial with sarilumab on hospital day two (illness day 15) for the management of COVID-19. The patient electively self-withdrew from the trial three days post-infusion (drug versus placebo) administration due to personal reasons, unrelated to any safety concerns.

The patient tested positive for COVID-19 via a nasopharyngeal swab polymerase chain reaction (PCR). Laboratory workup revealed a normal white blood cell count, with normal neutrophil and lymphocyte counts. However, mild thrombocytopenia and vitamin D deficiency were noted. He presented with mild to moderate elevation in C-reactive protein (CRP), ferritin, D-dimer, and interleukin-6 level. His procalcitonin level, however, was mildly elevated (Table 1).

**Table 1:** Lab values at admission

Laboratory test	Value at admission (reference range)
		C-reactive protein	12.09 mg/dL (<0.5 mg/dL)
D-Dimer	2.0 mg/L (<0.5 mg/L)
Ferritin	824 ng/mL (21.81–274.66 ng/mL)
Hemoglobin A1c	8.3% (4.0–6.5%)
Interleukin 6	59 pg/mL (<5 pg/mL)
Lactic acid	2.5 mmol/L (0.5–2.2 mmol/L)
Lactic dehydrogenase	398 μ/l (125–220 μ/L)
Platelet count	103 10^3^/μL (142.0–424.0 10^3^/μL)
Procalcitonin	0.13 ng/ml (0.0–0.07 ng/mL)
Troponin	0.013 (0.000–0.023)
Vitamin D	23.2 ng/mL (>30 ng/mL)
White blood cell	4.38 10^3^/μL (4.6–10.2 10^3^/μL)

**Figure 1:**
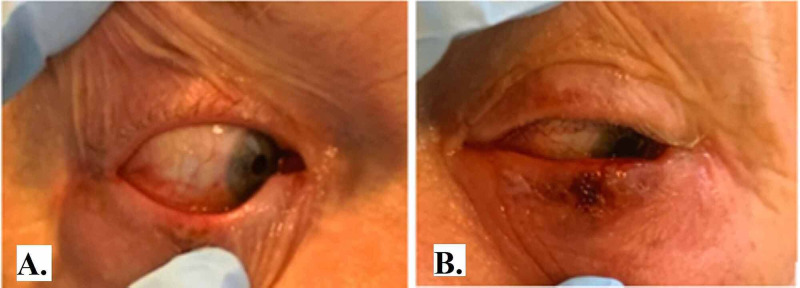
Right and left eye (panel A and B, respectively) showing bilateral periorbital and conjunctival erythema, and a shallow ulcer in the left lower eyelid anteriorly (panel B)

His chest X-ray at admission was unrevealing; however, a CT angiography (CTA) of his chest was negative for pulmonary embolism but revealed bilateral nodular and confluent sub-pleural and peri-bronchovascular ground-glass opacities. On the second day of admission (illness day 15), his left eye swelling and pain were noted to have worsened, with the development of a small ulcerative lesion anterior to the left lower eyelid (Figure 1).

**Figure 2:**
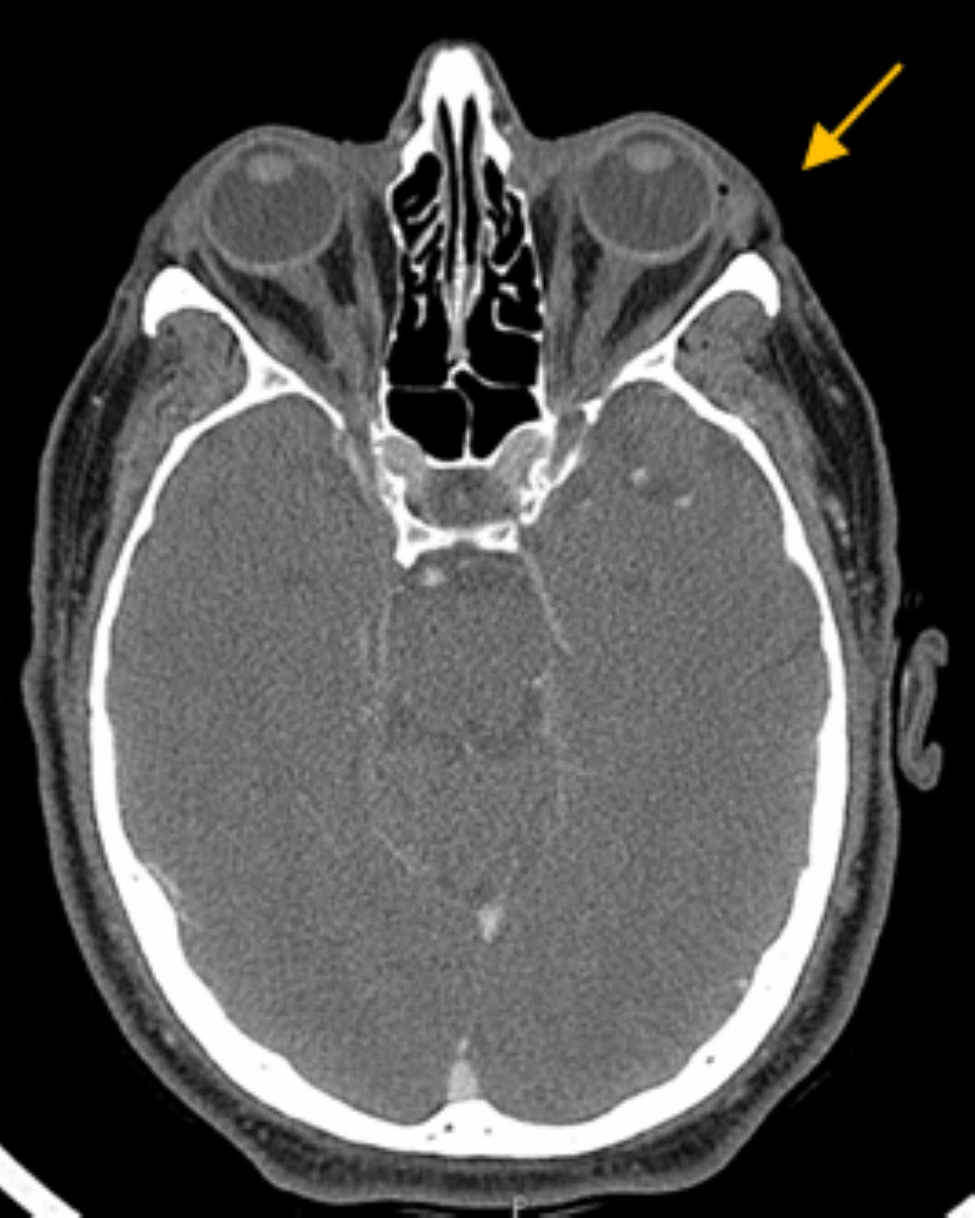
CT facial bones with contrast showing left-sided pre-septal cellulitis and probable subdermal fluid collection (yellow arrow) CT: computed tomography

A CT of facial bones showed a left-sided pre-septal and facial swelling consistent with cellulitis, and suggestive of a subdermal fluid collection measuring 1.5 x 0.4 x 0.9 cm, concerning for a superficial abscess (Figure 2). He was thus started on vancomycin and ceftriaxone.

His clinical presentation was most compatible with acute viral follicular conjunctivitis. For diagnostic purposes, conjunctival swabs were obtained from both eyes on hospital day seven (illness day 21), including a swab of the ulcer at the left lower eyelid. All the specimens were positive for HSV-1 PCR, and negative for severe acute respiratory syndrome coronavirus-2 (SARS-CoV-2), Adenovirus, and varicella-zoster virus (VZV) PCR.

His moderately severe SARS-CoV-2 infection was managed with a five-day course of hydroxychloroquine based on the limited evidence available at the time, in addition to supportive care. For the confirmed HSV-1 conjunctivitis, he was provided with a seven-day course of famciclovir and supportive care using artificial tears, cold compresses, and olopatadine hydrochloride ophthalmic solutions 0.1% every five to eight hours. The intravenous antimicrobial regimen targeting his pre-septal cellulitis was transitioned to oral cephalexin and doxycycline with instructions to complete 14 days of therapy.

During his inpatient stay, his oxygen requirements improved, along with his ocular symptoms. He was reached via telephone on post-discharge day six for continuity of care; he stated that he continued to notice some shortness of breath on exertion, though his ocular complaints had almost completely resolved.

## Discussion

Coronaviruses had been previously reported to be associated with conjunctivitis in humans but deemed to be mild and rare [4,5]. However, ocular complications of SARS-CoV-2 have not been widely reported. A study performed in the province of Hubei in China by Wu et al. found that 31.6% of COVID-19 patients (n=39) had ocular manifestations consistent with conjunctivitis. A meta-analysis conducted by Loffredo et al., including three studies of 1,167 patients with COVID-19, found that only 3% of patients with severe disease and 0.7% of patients with non-severe disease had conjunctivitis. In SARS-CoV-2 conjunctivitis, patients can present with ocular redness, irritation, foreign body sensation, tearing, and chemosis [2,5,6]. Our patient presented with all of the above characteristics. In addition, our patient did not experience blurred vision, which is consistent with other studies [2,5].

Clinical examination findings of SARS-CoV-2 conjunctivitis are mostly consistent with mild follicular conjunctivitis as observed in several other studies [4,5,7]. Similarly, our patient's exam findings were consistent with acute viral follicular conjunctivitis, likely related to COVID-19 with superimposed HSV-1 reactivation. The potential false-negative COVID-19 PCR test of the conjunctival swabs can be explained by poor sample collection, scarcity of viral RNA in conjunctival fluid, or test error. Of note, Wu et al. reported that only 16.7% of their COVID-19-positive patients under study had positive results for SARS-CoV-2 on PCR from both conjunctivae. This implies that perhaps not all COVID-19-related conjunctivitis will have positive SARS-CoV-2 PCR upon testing of conjunctival swabs. Conjunctivitis can rarely present as an initial manifestation of COVID-19, and ocular symptoms more commonly affect patients with severe systemic diseases [2,5,8]. Moreover, patients with ocular symptoms have been reported to present with higher white blood cell and neutrophil counts, higher levels of procalcitonin, CRP, and LDH compared to patients without ocular symptoms [2]. Our patient did have neutrophilia, elevated CRP, and LDH, but a mild elevation of procalcitonin, and his COVID-19 was deemed moderate in severity. Even though there is a low prevalence of SARS-CoV-2 nucleotides in tears, just like SARS-CoV-1, it is possible to transmit via the eyes [2,5]. Furthermore, in order to demonstrate that the genome detected corresponds to the infectious virus, Colavita et al inoculated Vero-6 cells with ocular samples positive for SARS-CoV-2 RNA, and cytopathic effects were observed five days post-inoculum [1]. It has been suggested that viral loads in conjunctival specimens gradually decrease over time, with less potential for transmissibility accompanied by improvement of the ocular symptoms [4]. Several authors have reported outcomes ranging from improvement to resolution of symptoms by the third week of illness [1,4]. Significantly, ocular manifestations of COVID-19 conjunctivitis are thought to be self-limited, and there are currently no reports of sight-threatening manifestations.

In our case, the patient had several risk factors for immunosuppression, including age, multiple comorbidities, and the use of antineoplastic therapy. Furthermore, we believe that multiple factors led to his HSV-1 reactivation, including recent use of the cytotoxic agent for his underlying lung malignancy, and his underlying SARS-CoV-2 infection. In addition, our patient had bilateral ocular disease, whereas HSV-1 conjunctivitis is almost always unilateral [9]. One hypothesis is that the initial COVID-19 conjunctivitis led to repeated eye-rubbing and trauma resulting in HSV-1 reactivation complicated by superimposed bacterial infection and autoinoculation of the other eye. Corneal staining with fluorescein was not performed for the evaluation of herpetic keratitis.

HSV conjunctivitis has been deemed to be the second most common cause of viral conjunctivitis after Adenovirus infection, and also the most serious one [6,9]. In one study conducted in a Minnesota county, the annual incidence of herpes eye symptoms was found to be 11.8 new cases for every 100,000 population [3]. With HSV, vesicles may appear on the face or eyelids and vision may be affected [6]. Supportive care for both COVID-19 and HSV-1 conjunctivitis are indicated, with the use of preservative-free artificial tears, antihistamine eye drops, and cold compresses. A short course of topical antibiotics can be added to prevent or treat bacterial superinfection [5]. For HSV conjunctivitis, topical antivirals like acyclovir have been used, and most patients achieve resolution within 14-30 days [6]. For our immunosuppressed patient, a systemic option was preferred over a topical antiviral, in addition to supportive care, yielding excellent results. Although atypical manifestations of COVID-19 infections including ocular manifestations were not looked into during the initial phase of the pandemic, it has now become critical to consider the possibility of COVID-19 infection in multiple organ systems, as well as the risk of co-infections including reactivation of viruses, based on the evolving evidence. This would tremendously help us in taking good care of these patients as well as to timely put preventative measures in place to control its spread both in the healthcare settings and among the population at large.

## Conclusions

Conjunctivitis is an atypical presentation of COVID-19 and can present with eye redness, ocular irritation, foreign body sensation, tearing, and chemosis. Detection of viral RNA in tears may not always be possible for diagnostic purposes. Nonetheless, COVID-19 conjunctivitis can in most instances be managed with a trial of frequent preservative-free artificial tears, cold compresses, and lubricating ophthalmic ointment. A short course of topical antibiotics can be added to prevent or treat bacterial superinfection. Lastly, HSV-1 should be on the differential diagnosis for any immunocompromised patient that presents with COVID-19 conjunctivitis.
